# Characteristics of Families with Members Going Out for Work and Associated Factors among Persons with Schizophrenia in Rural China

**DOI:** 10.3390/brainsci12111518

**Published:** 2022-11-09

**Authors:** Jia Cai, Yu-Jun Liu, Xian-Dong Meng, Yi Huang, Bo Liu, Mao-Sheng Ran

**Affiliations:** 1 Mental Health Center, West China Hospital, Sichuan University, Chengdu 610041, China; 2 Department of Social Work and Social Policy, Nanjing University, Nanjing 210023, China; 3 Jingzhou Mental Health Center, Jingzhou 434000, China

**Keywords:** family going out for work, schizophrenia, associated factors, rural China

## Abstract

Background: The characteristics associated with having family members going out for work among persons with schizophrenia in rural China are unknown. This study aimed to explore the characteristics of families with members going out for work and the risk factors among persons with schizophrenia in a rural area of China. Methods: This study employed a cross-sectional dataset from a mental health survey, using the International Classification of Disease, Tenth Revision (ICD-10), conducted among 152,776 people aged 15 years and older in Xinjin District, Chengdu, China, in 2015. Results: A total of 598 persons with schizophrenia were included in this study, and 20.4% (*n* = 122) of them had at least one family member who went out for work. Compared with those without family going out for work, participants with family going out for work had significantly larger numbers of family members, higher monthly incomes and lower percentages of social low-income insurance. Participants with family going out for work were more likely to be females, married and with higher levels of subjective and instrumental support. The statuses of families with members going out for work were significantly associated with larger numbers of family members and higher levels of instrumental support. Conclusions: Our findings indicate specific characteristics of families with members going out for work and factors associated with having family going out for work among persons with schizophrenia (number of family members and instrumental support). Culture-specific mental health policies and community-based services should be developed for persons with schizophrenia who have family going out for work in rural China.

## 1. Introduction

In the last three decades, China has undergone one of the largest rural-to-urban migrations in human history [1]. Although rural-to-urban migration is a common phenomenon in upcoming economies, China has been experiencing an unprecedented growth in this type of migration [1]. Amazingly, there were 292 million Chinese migrant workers in 2021 [2]. The rural-to-urban migration of family members has profound impacts on whole families within the Chinese population, especially those in rural China [3]. The majority of migrants are men, who seek higher wages in cities but leave their spouses, children and aging parents in rural villages [4]. Among these migrants, some are family of persons with schizophrenia.

Schizophrenia, a group of severe psychiatric disorders, is a persistent chronic disorder that imposes a severe economic burden on society and families [5]. Schizophrenia affects approximately 1% of people worldwide and has different prevalences (0.4–1.3%) in various areas and countries [6,7]. A review reported that annual costs for the schizophrenia population within a country ranged from USD 94 million to 102 billion [8]. Although the treatment of schizophrenia is mainly based on medication, 24% of persons with schizophrenia relapse within 1 year despite drug treatment [9]. The recovery of persons with schizophrenia is also related to other factors, such as degree of social support, family economic status, family interventions and family psychoeducation [10]. Evidence has also shown that the symptomatic remission of the disorder is associated with higher levels family-domain social support [11]. Family-based interventions (e.g., family psychoeducation) also have positive impacts on reducing the chance of psychotic relapse in schizophrenia. Our previous longitudinal study also indicated that, compared with those without family caregivers, persons with schizophrenia with family caregivers had better outcomes [12].

In China, the limited health resources mean that the burden of caregiving lies heavily within the family [13]. However, the treatment of persons with schizophrenia also places economic burdens on families [14]. In addition to emotional problems, most family members have been found to experience financial problems that affect their quality of life [15]. More importantly, the recovery of people with schizophrenia is closely linked to family circumstances, such as family economic status [16,17]. Therefore, some family of persons with schizophrenia have to go out to work during the period of urbanization in contemporary China. Previous studies have mostly focused on the mental health of migrant workers or their children [18]. However, up to now, no studies have been conducted on the characteristics and related factors of migrant family of persons with schizophrenia going out for work in rural China. A more comprehensive understanding of the family conditions of persons with schizophrenia, such as the characteristics of and the factors associated with family members going out for work, will be crucial for the development of health policies and mental health interventions for persons with schizophrenia.

What kinds of members of families of persons with schizophrenia will choose to go out to work? What factors will affect the choice of these family members to go out to work? No studies have been conducted to answer these questions. This study aimed to explore the characteristics of families with members going out for work and associated factors among persons with schizophrenia in rural China. We hypothesized that the status of families with members going out for work might be associated with number of family members, family economic status, severity of symptoms and social support for persons with schizophrenia.

## 2. Methods

### 2.1. Participants 

Data for this study were derived from the Chengdu Mental Health Project (CMHP), an ongoing longitudinal study on mental illness and mental health services in Xinjin District, Chengdu, China [12,19]. This study employed a cross-sectional dataset from the mental health survey that investigated 152 776 people aged 15 years and older in October 2015. In the survey in 2015, a total of 671 persons with schizophrenia were identified. Among these patients, 598 persons with schizophrenia (89.1%) who reported the situation of family members going out for work were included in this study. ‘Going out for work’ was defined as a family member going out to work for more than half a year in another county or city in China. Our previous publications contain more detailed information about this mental health survey [19,20,21,22]. In sum, all participants were identified through screening procedures for schizophrenia (e.g., trained investigators conducted face-to-face interviews with the head of each household and the key informants) and general psychiatric interviews. 

The inclusion criteria for this study included: (1) persons with schizophrenia identified according to the International Classification of Diseases, Tenth Revision (ICD-10) by a trained psychiatrist; and (2) persons with schizophrenia (or with the help of their family) completed the survey voluntarily. Subjects were excluded if they were ever diagnosed with head trauma; other major psychiatric disorders, such as substance use disorder; neurological diseases; or severe physical diseases. Trained psychiatrists conducted the diagnostic interviews. For participants with low literacy levels, the investigators read and explained the questionnaires. Participant social functioning and symptom severity were assessed by trained psychiatrists after interviews with the participants. 

This study complied with the content and requirements of the Helsinki Declaration and was reviewed and approved by the University Committee on Human Research Subjects. All research participants gave informed consent after a detailed explanation of the study. 

### 2.2. Measurements

The measurements in this study mainly included the following assessment scales and variables.

#### 2.2.1. Sociodemographic Characteristics

Participant characteristics used in this study included age, gender, education level, marital status, family size and family economic status. Family economic status was defined according to average family income, ≥ mean level or < mean level for the local community.

#### 2.2.2. Illness Characteristics

In this study, participant age of onset of mental disorders, duration of mental illness (i.e., the time from onset to current study), mental illness treatment status, relapse history, present mental status and work ability were measured. Measurement of going out for work relied on self-reporting by participants and reports from their families. 

#### 2.2.3. Social Functioning

The social functioning of the participants was measured by trained psychiatrists using the Global Assessment of Functioning (GAF) scale, which is validated and has been widely used in schizophrenia studies, including studies of persons with schizophrenia in China [21,23,24]. The total score range of the GAF is from 0 to 100; a higher score means better social functioning. 

#### 2.2.4. Duke Social Support Index (DSSI)

The DSSI was used to assess social support, including 23 items along three dimensions (social interaction (SI): 4 items; subjective social support (SSS): 7 items; and instrumental social support (ISS): 12 items). SI and SSS are designed with a 3-point Likert-type format, while ISS is dichotomous. Total scores range from 11 to 45, with higher scores reflecting more social support. DSSI was found to have a high internal consistency and Cronbach’s α in a Chinese study [25].

### 2.3. Statistical Analysis

In this study, missing data for numerical features were replaced by the mean values for the relevant features. The missing part of each categorical variable was included as a group in the analysis and none of the missing groups was significant in the final regression. Then, we used chi-square tests to compare the characteristics of groups of persons with schizophrenia with and without family going out for work. Student’s *t*-tests were used for continuous variables and chi-square tests were used for categorical variables. Finally, taking having family going out for work as the dependent variable, we conducted a multiple logistic regression analysis for each group separately. All the variables that showed significant differences (*p* < 0.05) between participants with or without family members going out for work in the previous comparison step were entered in the regression model to test for factors associated with going out for work. The SPSS (version 22.0) was used for statistical analyses.

## 3. Results

### 3.1. Demographic Characteristics of Participants

Table 1 shows the demographic characteristics of all the participants (*n* = 598). Among these, 52.8% were females, 79.6% had a low education level (less than 9 years), 55.7% were married and 72.6% had lower family economic status. Among all the participants, there were 122 families with members who once went out for work (20.4%), including parents (11, 9.0%), spouses (22, 18.0%), offspring (74, 60.7%) and others (15, 12.3%). 

There were no significant differences in age, education level or family economic status between participants with and without families going out for work (Table 1). Participants with family going out for work had significantly larger numbers of family members (*t* = 4.547, *p* < 0.001) and higher average monthly incomes (*t* = 4.733, *p* < 0.001) than those without family members going out for work. The results also showed that female participants had significantly higher rates of family going out for work than males (χ^2^ = 4.584, *p* < 0.05). Marital status was significantly different between participants with and without family going out for work. Participants with family going out for work had significantly higher rates of marriage (68.0%) and lower rates of divorce (4.1%) than those without family going out for work (52.5% and 12.6%, respectively). 

### 3.2. Clinical and Other Characteristics of Participants

As shown in Table 2, there were no significant differences in family history of mental illness, repeated relapse of mental illness, work ability, treatment status (never treated, taking antipsychotic drugs, being hospitalized and traditional medication), family caring attitudes, first age of onset and duration of illness between participants with and without family going out for work. The scores for the total GAF, family caring burden and social interaction also showed no significant differences between participants with and without family going out for work. However, compared with participants without family going out for work, participants with family going out for work had significantly lower rates of social low-income insurance (χ^2^ = 7.292, *p* < 0.01) and significantly higher levels of subjective support (*t* = 2.674, *p* < 0.01) and instrumental support (*t* = 2.345, *p* < 0.05). 

### 3.3. Factors Associated with Family Going Out for Work

As shown in Table 3, the results of the multiple regression analysis indicated that a large number of family members (OR = 0.779, 95% *CI* = 0.611–0.991, *p* < 0.05) and a higher score for instrumental support (OR = 0.913, 95% *CI* = 0.835–0.997, *p* < 0.05) were significantly associated with having family members going out for work among persons with schizophrenia. However, being female, with higher average monthly income, subjective support, social low-income insurance and being married were not included in the final model. 

## 4. Discussion

To the best of our knowledge, this is the first study to explore the characteristics of families with members going out for work and associated factors among persons with schizophrenia in rural China. This study showed that 20.4% of persons with schizophrenia had at least one family member going out for work. This study arguably contributes rigorous empirical data to the existing knowledge of characteristics of families with members going out for work and associated factors among persons with schizophrenia in low- and middle-income countries (LMICs). The strength of this study is the use of a relatively large representative community sample in a Chinese rural area [20,21]. 

In this study, we found significant differences in sociodemographic characteristics between participants with and without family members going out for work. Participants with family going out for work had significantly larger numbers of family members—a significant factor associated with family going out for work. It is not difficult to understand that having more family members means more opportunities for family members other than the primary caregiver to work outside the home in a rural area. Our previous study also found that a larger number of family members made it more possible to retain at least one family caregiver over a long period of time [12]. This is crucial for the treatment and rehabilitation of persons with schizophrenia in rural communities [9]. In addition, higher average monthly income and lower rate of social low-income insurance were significantly associated with the status of families with members going out for work in the univariate analysis, even though the results were not maintained in the final regression model. Previous studies have reported a strong correlation between patient recovery and family burden, especially family economic burden [26]. Our earlier studies also indicated that low family economic status is a predictive factor for poor long-term outcomes among persons with schizophrenia in rural communities [27]. As the status of families going out for work may play an important role in increasing family income, reducing family economic burden and improving the treatment and outcome of persons with schizophrenia, further studies should be conducted to explore the relationship between the status of families with members going out for work and the outcomes of persons with schizophrenia. 

The findings of this study also showed that, compared with participants without family going out for work, participants with family going out for work were significantly more likely to be female and have higher rates of marriage and higher scores for subjective support and instrumental support. Among these factors, higher score for instrumental support was a significant factor in the regression model associated with having family going out for work. There are several reasons that may account for these differences. Firstly, previous researches have reported that gender-related differences (e.g., hormonal effects, gene dosage effects, epigenetic effects, etc.) might protect women, even during embryonic development, such that males with schizophrenia have earlier onsets of the disease and manifest more severe neurodevelopmental forms of illness [28,29]. As a result, males with schizophrenia have higher rates of negative symptoms and poorer disease prognosis [30]. Contrastingly, females with schizophrenia generally exhibit more favorable clinical outcomes in terms of disease course than males with schizophrenia, as reflected in lower relapse rates, higher remission rates and a lower likelihood of being admitted to hospital [31,32]. Females with schizophrenia are more likely to be accepted by their families and communities, especially in rural areas, than males with schizophrenia [31,32]. Therefore, caring for males with schizophrenia imposes a heavier caregiving burden on family caregivers due to their poorer social functioning and violent behavior [33]. Given the lighter burden of caring for females with schizophrenia, the families of such persons with schizophrenia are more likely to go out for work to earn more money.

Secondly, evidence showed that, compared with unmarried persons with schizophrenia, married individuals might have stronger social networks and higher levels of support, better quality of life [34] and lower risks of committing crime and attempting suicide [35]. The results of this study showed that most family members going out for work were the offspring and spouses of persons with schizophrenia (60.7% and 18.0%, respectively). In rural China, due to limited community mental health services, the parents, offspring and spouses of persons with schizophrenia are more likely to be the primary caregivers. A long and stable marriage in persons with schizophrenia plays an important role in the full or partial remission of the illness and in having a better ability to engage in employment [36]. Moreover, this study also showed that persons with schizophrenia with family members going out for work had higher levels of subjective support and instrumental support. Thus, the results of this study indicate that marriage and family and social support (subjective and instrumental support) are crucial for having family go out for work.

The findings from this study indicated that having a larger number of family members and a higher score for instrumental support were significant factors associated with having family members going out for work among persons with schizophrenia in the final regression model. As family members are more likely to be the primary caregivers of persons with schizophrenia in rural China, only larger numbers of family members make it possible for family to go out for work in other places. Moreover, the results of this study also indicate that families with higher levels of instrumental support, not only subjective support, are more likely to have members go out for work. Therefore, this study indicates that family are more likely to go out for work only if there are more family members and there is the necessary instrumental support to help the family (from other relatives or neighbors) [37,38]. Moreover, psychosocial interventions are needed in persons with schizophrenia, these playing an important role in improving functional outcomes and quality of life and reducing relapse frequencies [10,39]. The authors of this study suggest that government and community organizations and mental health professionals should provide necessary support and services (social welfare, health services and management, psychosocial intervention, etc.) for migrants with relatives and the families of persons with schizophrenia in rural areas [40].

In addition, it is worth mentioning that clinical factors, such as duration of illness, GAF score and the treatment (e.g., antipsychotic medication, being hospitalized) of persons with schizophrenia, were not significantly associated with having family members going out for work in this study. The result indicates that the clinical situations of persons with schizophrenia (e.g., duration of illness, symptoms, functioning and treatments) may not be the major factors for families in making decisions to go out for work. However, the result may have been impacted by the lower rates of antipsychotic medication and hospitalization for persons with schizophrenia in rural China [41]. The mean value of ‘first age of onset’ was relatively high in this study. Possible reasons for this may include: (1) the special situation in current Chinese rural areas of many young people (maybe including some young people with schizophrenia) going out for work in urban areas; and (2) potential recall bias of the first age of onset [21,42]. The impact of the stigma of mental illness on the families with members going out for work has not been explored in this study [20,42]. Further studies should be conducted in these areas. 

Given the relatively large representative community sample in a Chinese rural area, our findings may be generalizable to populations of persons with schizophrenia in other rural areas and even other LMICs that have similar socioeconomic environments. 

### Limitations of the Study

This study has the following limitations. First, this study was a cross-sectional investigation and could not explore the causal relationships between family members going out for work and other factors. Longitudinal studies should be conducted in the future to explore the causal associations. Second, reports of families with members going out for work were based on the participants’ self-reports, which may have resulted in underestimation. Third, as the mean age of the participants in the study was over 50 years, the conclusion might not be representative of younger persons with schizophrenia. Fourth, this study was conducted in a rural area of China, and the results might not be applicable to urban regions.

## 5. Conclusions

It is crucial to understand the characteristics of families with members going out for work and associated factors among persons with schizophrenia in rural areas, especially during the period of social change (urbanization, migration) in contemporary China. This study has indicated that number of family members and instrumental support are major factors associated with having family members going out for work. Culture-specific mental health policies and community-based services (guidance for migration, community mental health care, family support, etc.) should be developed for persons with schizophrenia who have family going out for work in rural China. This study is also crucial for the development of public health policies and services for migrants with relatives with schizophrenia in rural areas.

## Figures and Tables

**Table 1:** Demographic characteristics of participants with and without family going out for work.

	Total (*n* = 598, 100%)	With Family Going Out for Work (*n* = 122, 20.4%)	Without Family Going Out for Work (*n* = 476, 79.6%)	t/χ^2^
Gender				4.584 *
Female	316 (52.8)	75 (61.5)	241 (50.6)	
Male	282 (47.2)	47 (38.5)	235 (49.4)	
Education level				0.985
≤9 years	476 (79.6)	98 (80.3)	378 (79.4)	
>9 years	122 (20.4)	24 (19.7)	98 (20.6)	
Marital status				16.374 **
Unmarried	116 (19.4)	15 (12.3)	101 (21.2)	
Married	333 (55.7)	83 (68.0)	250 (52.5)	
Divorced	65 (10.8)	5 (4.1)	60 (12.6)	
Widowed	73 (12.3)	15 (12.3)	58 (12.2)	
Other	11 (1.8)	4 (3.3)	7 (1.5)	
Family economic status				3.137
Above average	17 (2.8)	6 (4.9)	11 (2.3)	
Medium	147 (24.6)	33 (27.1)	114 (24.0)	
Lower than average	434 (72.6)	83 (68.0)	351(73.7)	
	Mean (SD)	Mean (SD)	Mean (SD)	
Age (years)	54.59 (14.52)	53.30 (14.76)	55.01 (14.28)	−1.177
Number of family members	2.95 (1.40)	3.47 (1.31)	2.82 (1.43)	4.547 ***
Average monthly income	2049.49 (1980.23)	2743.91 (2056.65)	1702 (1702.11)	4.733 ***

* *p* < 0.05, ** *p* < 0.01, *** *p* < 0.001.

**Table 2:** Clinical and other characteristics of participants with and without family going out for work.

	Total (*n* = 598, 100%)	With Family Going out for Work (*n* = 122, 20.4%)	Without Family Going out for Work (*n* = 476, 79.6%)	t/χ^2^
Family history of mental illness, positive	104 (17.4)	25 (20.5)	79 (16.6)	0.256
Repeated relapse of mental illness	449 (75.1)	96 (78.7)	353 (74.2)	1.065
Work ability				5.453
Able to work	149 (24.9)	40 (32.8)	109 (22.9)	
Partly able to work	309 (51.7)	54 (44.3)	255 (53.6)	
Unable to work	140 (23.4)	28 (22.9)	112 (23.5)	
Never treated	121 (20.2)	27 (22.1)	94 (19.8)	0.341
Taking antipsychotic drugs	164 (27.4)	37 (30.3)	127 (26.7)	0.649
Being hospitalized	16 (2.7)	4 (3.3)	12 (2.5)	0.214
Traditional medication	29 (4.9)	7 (5.7)	22 (4.6)	0.262
Family caring attitudes				0.353
General	538 (90.0)	108 (88.5)	430 (90.3)	
Poor or maltreatment	60 (10.0)	14 (11.5)	46 (9.7)	
With social low-income insurance	188 (31.4)	26 (21.3)	162 (34.0)	7.292 **
				
	Mean (SD)	Mean (SD)	Mean (SD)	
First age of onset (years)	32.96 (13.35)	32.90 (14.37)	33.23 (13.13)	−0.231
Total duration of illness (years)	20.53 (13.23)	19.59 (13.16)	20.55 (13.29)	−0.684
Total GAF score	61.36 (31.10)	62.62 (19.15)	61.00 (34.74)	0.479
Family caring burden score	22.64 (5.73)	21.64 (4.87)	22.80 (6.02)	1.492
Social interaction score	6.92 (2.01)	7.31 (2.05)	6.86 (1.99)	1.760
Subjective support score	14.47 (3.88)	15.86 (4.24)	14.24 (3.83)	2.674 **
Instrumental support score	19.88 (4.54)	21.13 (3.81)	19.56 (4.66)	2.345 *

GAF: Global Assessment Function. * *p* < 0.05, ** *p* < 0.01.

**Table 3:** Multivariate logistic regression of influencing factors associated with having family going out for work.

	Schizophrenia		
	Wald	OR	95% CI
Large number of family members	4.125	0.779	0.611–0.991 *
Instrumental support score	4.063	0.913	0.835–0.997 *
Being female	3.105	1.998	0.925–4.313
Higher average monthly income	0.766	0.999	0.867–1.159
Subjective support score	0.418	0.963	0.858–1.080
With social low-income insurance	0.281	1.261	0.536–2.958
Married	0.060	1.061	0.660–1.707

OR: adjusted odds ratio; CI: confidence interval. * *p* < 0.05.

## Data Availability

The de-identified data are available on reasonable request to the corresponding author.
